# Nest microclimate during incubation affects posthatching development and parental care in wild birds

**DOI:** 10.1038/s41598-019-41690-4

**Published:** 2019-03-26

**Authors:** Alexander J. Mueller, Kelly D. Miller, E. Keith Bowers

**Affiliations:** Department of Biological Sciences and Edward J. Meeman Biological Station, University of Memphis, Memphis, TN USA

## Abstract

It is widely accepted that recent increases in environmental temperature have had a causal effect on changing life histories; however, much of the evidence for this is derived from long-term observations, whereas inferences of causation require experimentation. Here, we assess effects of increased environmental temperature during incubation on posthatching development, nestling begging and parental care, and reproductive success in two wild, cavity-nesting songbirds, the Carolina wren and prothonotary warbler. We heated experimental nests only during incubation, which increased nest-cavity temperature by ca. 1 °C. This reduced the length of the incubation and nestling periods, and reduced fledging success in prothonotary warblers, while nestling Carolina wrens had similar fledging success but reduced body condition in response to increased temperature. Increased nest-cavity temperature during incubation also reduced posthatching begging by nestlings generally and parental care within Carolina wrens specifically, suggesting potential mechanisms generating these carry-over effects. Offspring body mass and fledging age are often predictive of post-fledging survival and recruitment. Thus, our results suggest that increasing temperatures may affect fitness in wild populations in species-specific ways, and induce life-history changes including the classic trade-off parents face between the size and number of offspring.

## Introduction

Recent environmental change, including shifts in temperature, have affected the life histories of various organisms. Such effects include shifts in the timing of migration and breeding phenology for various avian species^1–5^, and understanding the mechanisms underlying these responses has emerged as a major aim of research in the evolution of life histories^6–9^. One direct effect of temperature includes that on parental incubation effort and embryonic development. Incubation of eggs is generally thought to be costly^10–13^, which might otherwise favour parents that incubate less; however, incubation is a critical component of parental care^13–15^, as offspring will not develop without the appropriate amount of incubation from their parents. Thus, incubation effort may be subject to opposing selective forces that favour a parental optimum somewhere between extremes. Exactly where this optimum lies may depend on ambient temperature, as the extent of maternal effort dedicated to incubation is partly dependent on nest microclimate^10,16–18^. For example, female tree swallows vary incubation constancy and duration of off-bouts, or the time spent off the nest, in relation to environmental temperatures^19^. When incubating in a relatively warmer environment, female birds could maintain greater body condition, which might augment incubation effort^16,20,21^, or potentially ameliorate the costs of incubation to parents^10,11^.

The length of the incubation period is often negatively correlated with environmental temperature^5,17,19,22–26^. This may arise as (i) a direct effect of ambient temperature on the rate of development, for example by reducing the rate at which eggs cool during parental recesses from incubation, known to have carry-over effects on postnatal development^24,27^, as (ii) a consequence of temperature affecting parental incubation behaviour, or (iii) a combination of both. Carry-over effects occur whenever conditions experienced during one life-history stage affect performance or behaviour in subsequent stages^28–30^, and there is evidence that environmental temperature during development can induce these kinds of effects. In some species, parents modify their incubation behaviour according to the temperature of the nest microclimate^10,31^, which may allow for the maintenance of a relatively constant incubation temperature regardless of environmental conditions while parents are on the nest incubating their eggs, but incubating parents routinely take breaks, leaving eggs unattended and, thus, susceptible to environmental temperatures. Such variation in environmental temperature may affect components of postnatal development^18,27,28,32–34^, and recent work has revealed potential mechanisms that might mediate effects of environmental temperature on postnatal behaviour^35,36^. Thus, increases in temperature may not only accelerate development within the egg, but may also induce additional phenotypic changes that ultimately shape offspring survival and fitness^15,37–39^. For example, hatching success has been found to increase with warmer incubation temperatures^24,40^, but an optimal temperature has also been observed in a number of species, as increasing temperatures can negatively affect measures of nestling condition and immunocompetence^41,42^. Indeed, even slight variations (<1.0 °C) in either incubation temperature or the temperature of the nest microclimate prior to hatching can induce carry-over effects on nestling development^41,43^. It is important to note, however, that effects of increasing environmental temperature may be species-specific^27^, as some studies have reported effects on offspring that are apparently beneficial while others appear putatively harmful^24,40–42^. Thus, the generality of these effects and their consequences at population and community levels remain unclear.

Although increased nest temperature might be beneficial under low average environmental temperature in early spring (e.g., possibly by reducing the cost of incubation to parents), the effects of nest microclimate on the duration of incubation may also carry over to generate costs to offspring in subsequent life-history stages. For example, a recent analysis of a 36-year data set from a study of a population of house wrens^5^ revealed an advance in breeding phenology in the population associated with increasing temperatures since 1980, and that incubation periods had shortened over this time as well, as there was a negative correlation between ambient temperature and the duration of incubation. That study also revealed that nestling periods had lengthened during warmer breeding seasons, as incubation duration and the period of nestling growth were negatively correlated. However, it is important to note that these recent findings are observational, and may be influenced by additional, unexplored variables. For example, the increase in the length of the nestling stage since 1980 may reflect a direct effect of reduced incubation duration on the developmental state and posthatching growth of nestlings, but could also be caused by a reduction in the quantity or quality of prey with increasing temperatures over this time, thereby prolonging nestling growth independent of incubation duration. Thus, while long-term observations are essential, characterizing the causal effects of environmental change on life histories ultimately requires an experimental approach.

In this study, we experimentally manipulated the temperature of the nest microclimate during incubation to investigate whether increasing temperature affects both incubation duration and posthatching development. We tested this in two cavity-nesting woodland birds, the Carolina wren (a non-migratory, year-round resident) and the prothonotary warbler (a trans-Gulf migrant that winters in Central America and northern South America). We heated experimental nests using battery-powered resistive heating coils, which successfully led to a subtle increase in the temperature of nest cavities. However, the nests built by our two study species differ markedly, with the wrens building a dome composed of leaves and moss that surrounds the eggs, and warblers building a more open cup within the nest cavity (i.e., eggs are fully exposed and not insulated by moss or other materials during incubation recesses). Thus, we tested whether increases in temperature of the nest microclimate during incubation affected these clutches in a species-specific way. We predicted that, overall, the increased temperature would reduce the time required for eggs to hatch, and that this would affect the period of nestling development, possibly by delaying fledging age^5^.

An effect of environmental temperature during incubation on fledging age could be manifest directly on offspring, indirectly through parental behaviour, or a combination of both. For example, in addition to parental incubation behaviour varying with environmental temperature, there is evidence that the temperature at which offspring develop has organizational effects on their neuroendocrine systems and stress reactivity^35,36^. These may also influence nestling begging solicitations and, consequently, posthatching parental care^44–46^, thereby providing a potential mechanism through which temperature carries over to affect postnatal behaviour and performance. As such, we also assessed effects of increasing temperature on maternal incubation behaviour^10,16–20^, and posthatching parental care and offspring development. Variation in the temperature of the nest microclimate is known to induce carry-over effects on nestling development and physiology posthatching^27,35,36^. We predicted that any such carry-over effects might also be associated with patterns of parental care and the soliciting of care by nestlings. Finally, if changes in temperature induce changes in the duration of parental care for nest-bound young, we predict that patterns of variation in parental care that are attributable to changes in temperature during incubation should also be associated with variation in parents’ future breeding success. Thus, we assessed potential consequences for breeding success and the return of adults to be breeding population the following year.

## Results

### Effect of manipulation on nest microclimate

As expected, experimental nest microclimates were significantly warmer, on average, than control nests from 7:00–13:00 (i.e., the time during which heating coils were active; mean ± SE; experimental: 24.8 ± 0.14 °C, control: 23.8 ± 0.14 °C; *F*_1,45_ = 24.33, *P* < 0.0001; see Supplementary Fig. S1). Temperature differentials between internal and external iButton measures confirmed that experimental nest microclimates were warmer than control nests relative to ambient conditions outside the nestbox (*F*_1,45.1_ = 21.63, *P* < 0.0001; see Supplementary Fig. S2a), and temperature was marginally but non-significantly elevated when analysing temperature averaged over the entire day (*F*_1,45_ = 4.02, *P* = 0.0510; see Supplementary Fig. S2b). Thus, the manipulation resulted in a subtle, yet noticeable, increase in environmental temperature within nest cavities.

### Effects on incubation behaviour

Despite the increase in temperature within experimental nests, there was no effect of treatment on incubation constancy or the number of on/off bouts per hr (*F*_2,40_ = 0.41, *P* = 0.6683), nor did treatment and species interact to affect these behaviours (*F*_2,39_ = 0.72, *P* = 0.4945). Nonetheless, there were marked differences between species (*F*_2,40_ = 9.76, *P* = 0.0004) in both the proportion of time spent incubating (least-squares mean ± SE: Carolina wrens: 0.908 ± 0.040, prothonotary warblers: 0.720 ± 0.031) and in the number of on/off bouts per hr (least-squares mean ± SE: Carolina wrens: 1.206 ± 0.161, prothonotary warblers: 2.122 ± 0.124). Although there was no evidence for a treatment effect on incubation behaviour *per se*, we also tested whether incubation behaviour might be correlated with environmental temperature (i.e., as a continuous predictor), and detected a significant association between temperature and incubation behaviour (*F*_2,37_ = 4.26, *P* = 0.0216), indicating that warmer nests were associated with a reduction in the number of on/off bouts.

### Duration of incubation and the nestling stage

**Figure 1:**
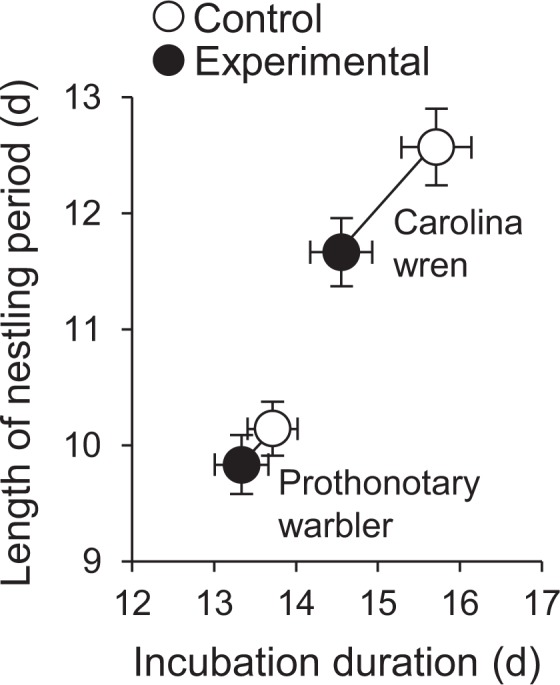
Incubation duration and length of the nestling stage. Plotted are least-squares means ± SE.

The duration of both the incubation and nestling stages was reduced by the increase in temperature in the nest cavity during incubation (Table 1; Fig. 1). There was no interaction between treatment and species in their effect on these variables (*F*_2,36_ = 2.32, *P* = 0.1130), indicating that both incubation duration and fledging age were advanced by the increase in temperature in both species. Standardized canonical coefficients (Table 1) indicated that the overall treatment effect was driven by effects on both incubation and nestling stages; that these coefficients are of the same sign indicates a positive correlation between incubation duration and the length of the nestling stage across treatments. However, incubation duration and the length of the nestling stage also varied with clutch-initiation date, but in different directions; that the standardized canonical coefficients for the effect of clutch-initiation date are of opposite sign (Table 1) indicates a negative correlation between incubation duration and the length of the nestling stage over the course of the breeding season. In other words, as the breeding season progressed, incubation duration declined (correlation with clutch-initiation date: *r*_40_ = −0.39, *P* = 0.0119), while the length of the nestling period increased (correlation with clutch-initiation date: *r*_40_ = 0.48, *P* = 0.0014).

**Table 1:** Effects of incubation treatment, species, and time of year on the duration of incubation and fledging age, and effects of incubation treatment, species, and begging frequency on posthatching parental care.

Summary of effects on incubation duration and the length of the nestling stage						
Source of variation	*F*	df	*P*	Standardized Canonical Coefficients		
				Incubation		Nestling stage
Treatment	6.43	2, 37	0.0040	1.136		1.139
Species	57.75	2, 37	<0.0001	0.999		1.291
Clutch-initiation date	5.18	2, 37	0.0104	1.347		−0.889
**Summary of effects on posthatching parental care**						
**Source of variation**	***F***	**df**	***P***	**Standardized Canonical Coefficients**		
				**Maternal provisioning**	**Maternal brooding time**	**Paternal provisioning**
Treatment	1.04	3, 37	0.3866	0.789	−0.126	0.694
Species	1.10	3, 37	0.3601	−0.364	1.125	0.146
Begging frequency	8.96	3, 37	0.0001	0.395	1.354	0.320
Treatment × species	3.97	3, 37	0.0150	0.148	1.411	−0.137

Both analyses are multivariate linear models assessing sources of variation in incubation duration and the length of the nestling stage as dependent variables, and in maternal provisioning and brooding time and paternal provisioning as dependent variables.

### Posthatching development and parental care

**Figure 2:**
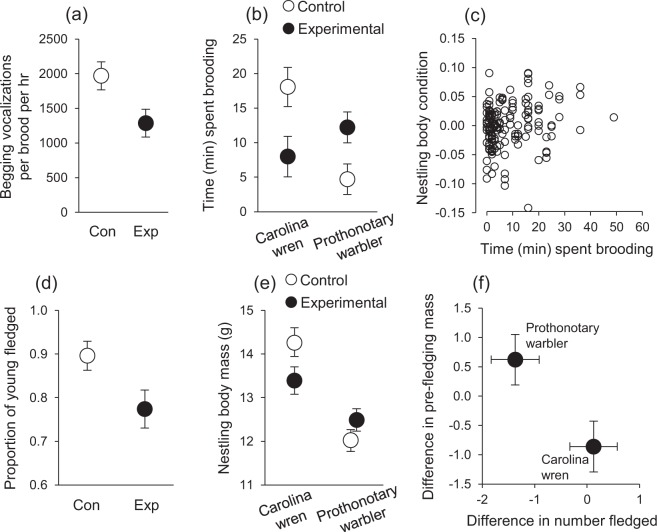
(**a**) Nestling begging vocalizations four days posthatching, observed during our observations of parental care. (**b**) Species-specific effects on the amount of time females spent brooding their young. (**c**) Effect of maternal brooding time on nestling pre-fledging body condition across both species (which were not affected differently by the treatment). Body condition here is calculated as the residual of a log_10_(mass) × log_10_(tarsus) linear regression for graphical purposes. (**d**) Number of young fledged, (**e**) pre-fledging body mass by species and treatment, and (**f**) the effect of experimentally increased environmental temperature on these variables (i.e., relative to control nests) for each species.

The proportion of eggs laid that subsequently hatched was not affected by our treatment (*F*_1,41_ = 0.17, *P* = 0.6798), species (*F*_1,41_ = 0.88, *P* = 0.3536), nor by a treatment × species interaction (*F*_1,40_ = 2.01, *P* = 0.1645). However, the experimental increase in environmental temperature during incubation carried over to affect nestling development after hatching. Nestlings that developed as embryos in environments characterized by increased temperatures solicited food from their parents at a lower rate than control nestlings (*F*_1,39_ = 5.77, *P* = 0.0212; Fig. 2a), while controlling for variation in hatching date (*F*_1,39_ = 0.24, *P* = 0.6288), brood size (*F*_1,39_ = 0.64, *P* = 0.4299), and species (*F*_1,39_ = 6.74, *P* = 0.0132). Higher nestling begging rates were associated with increased parental brooding and feeding (Table 1). That the canonical coefficients for begging frequency (Table 1) are all of the same sign indicates a positive correlation among the dependent variables across a range of nestling begging rates. There was also a significant interaction between treatment and species in their effect on parental care, manifest primarily through maternal brooding time (Table 1). Follow-up tests to tease apart this interaction revealed that, within the Carolina wrens, experimental females spent significantly less time brooding their young after hatching than control females (*F*_1,37_ = 6.09; *P* = 0.0181; Fig. 2b), whereas within the warblers, experimental females spent more time brooding their young than controls (*F*_1,37_ = 5.82; *P* = 0.0206; Fig. 2b). No other differences within species in maternal or paternal care were significant (all *P* > 0.05). Maternal brooding time had the greatest effect on nestling condition (i.e., size-adjusted body mass) when measured prior to fledging (estimate ± SE = 0.029 ± 0.013, *F*_1,37_ = 4.69, *P* = 0.0369; Fig. 2c), but neither maternal nor paternal provisioning had any effect on pre-fledging condition (both *P* > 0.25).

### Fledging success and nestling condition

Although there was no difference in clutch size (*F*_1,44_ = 0.98, *P* = 0.3284) or hatching success (above) between control and experimental nests, experimental nests produced fewer fledglings per egg laid than control nests (Table 2; Fig. 2d). We also detected an interaction between treatment and species in their effect on nestling pre-fledging condition (Table 2; Fig. 2e). Follow-up tests revealed that Carolina wren nestlings in experimental nests were in poorer condition, on average, than were control nestlings (*F*_1,35.6_ = 4.19, *P* = 0.0482; Fig. 2e), while there was no effect of incubation treatment on pre-fledging body condition in prothonotary warblers (*F*_1,36_ = 1.95, *P* = 0.1707). There was no effect of the treatment nor a treatment × species interaction on nestling tarsus length (treatment: *F*_1,36_ = 1.03, *P* = 0.3179; treatment × species: *F*_1,35.9_ = 0.02, *P* = 0.8979), suggesting that the effect on nestling condition was manifest by differences in mass gained by nestlings and not by any differences in skeletal size. Thus, the effect of increased environmental temperature on the trade-off between offspring number and condition differed for the two species (Fig. 2f).

**Table 2:** Effects on the number of young fledged per egg laid, and pre-fledging body mass of nestlings.

Source of variation	*F*	df	*P*
**Effects on fledging success**			
Treatment	4.98	1, 41	0.0311
Species	2.19	1, 41	0.1464
**Effects on pre-fledging body mass**			
Treatment	0.55	1, 35.9	0.4626
Tarsus length	159.64	1, 134.0	<0.0001
Species	20.07	1, 67.6	<0.0001
Banding date	5.27	1, 36.2	0.0276
Treatment × species	6.13	1, 35.6	0.0182

Fledging success was analysed using a generalized linear model assessing the number of young fledged in “successes/trials” syntax (see methods). Pre-fledging body mass was analysed using a linear mixed model with nest identity as a random effect to account for the non-independence of siblings within broods (the random effect of brood explained 11.8% of the variation observed in nestling mass).

### Effects on the probability of breeding in future years

**Figure 3:**
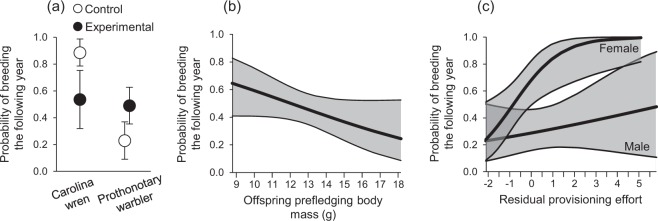
Probability of adults breeding at the study site in the year following our manipulation. (**a**) Species-specific effect of treatment, (**b**) effect of nestling pre-fledging body mass (a measure of parental investment), and (**c**) sex-specific effect of residual provisioning effort (i.e., responsiveness to nestling begging vocalizations). Plotted in (**a**) are least-squares means ± SE (adjusted for other terms in the model), and in (**b**,**c**) is the probability of breeding ± 95% CL.

A number of factors affected the probability of parents’ breeding in their study populations in the year following our manipulation, including species- and sex-specific effects of our treatment and parental investment (Table 3; Fig. 3). Specifically, the effect of our experimental manipulation on adult return rates was species-specific (Table 3; Fig. 3a) and driven by a slight reduction in breeding probability for experimental Carolina wrens relative to controls, but no effect on the warblers (Fig. 3a). Parents fledging heavier nestlings were less likely to return to breed (Fig. 3b). This effect was not caused by between-species differences in either size or return rates, as the negative relationship was apparent within both species individually (species × nestling body mass interaction: *P* > 0.9). There was also a sex-specific effect of parents’ residual provisioning effort (i.e., the degree to which they responded to changes in nestling begging), such that parents who were more responsive to nestling begging solicitations were generally more likely to return to breed in the subsequent breeding season overall, but this effect was much stronger for females than for males (Table 3; Fig. 3c), an effect that was apparent in both species.

**Table 3:** Probability of parents breeding in the subsequent year.

Source of variation	Estimate ± SE	χ^2^	df	*P*
Treatment	−1.17 ± 0.82	0.39	1	0.5332
Species	−0.18 ± 1.17	2.27	1	0.1323
Sex	1.87 ± 0.57	9.73	1	0.0018
Residual provisioning	0.06 ± 0.19	6.69	1	0.0097
Offspring pre-fledging mass	−2.10 ± 1.07	4.69	1	0.0303
Brood size	−4.88 ± 3.19	2.48	1	0.1155
Pre-fledging mass × Brood size	0.38 ± 0.24	2.70	1	0.1005
Treatment × Species	3.08 ± 1.51	5.09	1	0.0240
Residual provisioning × Sex	1.15 ± 0.51	7.03	1	0.0080

Return rates were analysed using a generalized linear model with a binary outcome (1 = returned; 0 = did not return).

## Discussion

Our manipulation caused an ecologically relevant increase in the temperature of the nest microclimate that extended peak daytime temperatures into the morning hours, consistent with recent and forthcoming climatic changes. On average, temperature differentials did not exceed 2 °C for experimental nests during the time that our heaters were active. Although we observed no effect on hatching success between groups, the subtle, yet significant, increase in temperature during incubation led to changes in nestling development and posthatching survival.

Not unexpectedly, we found that increasing temperature of the nest microclimate shortens the duration of embryonic development overall, consistent with previous results from both observational and experimental studies^5,17,19,22^. We also predicted that increases in environmental temperature may influence the duration of the nestling stage; in a recent observational study, while the increase in environmental temperature that had occurred over recent decades led to a shortening of the incubation period, the length of the nestling stage had grown, and the duration of the incubation and nestling stages were negatively correlated^5^. This may be costly in natural populations if the prolonged time required for nestling development leads to increased probability of depredation or parasitism^47–50^, or reproductive costs to parents associated with extended postnatal care. We predicted that this negative correlation might be reflective of constraints on nestling development induced by the rate of embryonic development (e.g., shorter incubation times could necessitate longer nestling periods; *sensu* ref.^29^). This was not the case, however, in our experimental study, which revealed a positive correlation between these variables. Thus, the negative correlation observed over several decades by Bowers *et al*.^5^ might not be a direct effect of incubation duration on nestling development, but an indirect effect mediated by an unexplored third variable, such as changes in food availability over time that prolong posthatching development before nestlings are able to fledge^51–53^. Results of the current study suggest that, by reducing incubation duration, increased temperatures may reduce the amount of parental effort required for incubation, thereby enhancing components of posthatching parental care and allowing parents in warmer environments to fledge their young at an earlier age. This suggests that incubation is indeed costly and that manipulating the temperature of the nest microclimate could be a useful context in which to study trade-offs between investment in incubation and other components of the nesting cycle^10,16–18,32,54^.

Shorter nestling periods might have a number of consequences. Fledging at younger ages could potentially benefit parental survival by reducing energy allocated to provisioning nest-bound young, allowing parents to increase their investment in self-maintenance, but fledging at a premature developmental stage reduces offspring recruitment generally^53,55^, thereby reducing parental fitness. Despite the potential reduction in offspring survival imposed by fledging at too early of age, this detriment to parents’ inclusive fitness might be overcome in multibrooded species if parents are able to produce a subsequent brood earlier within the season. Typically, offspring that fledge from the nest earlier within a breeding season have increased recruitment as breeding adults in local populations relative to nestlings produced later in the season^5,56–59^, an effect that appears to be generally stronger than that of early fledging age. A higher abundance of arthropod prey, along with more time to mature before autumn migration or winter, might provide earlier-fledged offspring advantages over those that fledge later in the season. Whether these hypotheses are true, however, awaits further study.

One major aim of our study was to test for carry-over effects of temperature during incubation on posthatching development, and we found experimental nestlings in both species to beg for food at a reduced rate. Intriguingly, hatchling wood ducks incubated at higher temperatures were recently found to be less proactive than those incubated at lower temperatures^35^, which might help explain, at least in part, the effect we observed on nestling begging and posthatching development. Indeed, recent experiments reveal an important role for temperature in directly shaping avian neuroendocrine systems^35,36^. There was no effect of incubation treatment on fledging success in Carolina wrens, yet offspring pre-fledging body condition, an indication of phenotypic “quality”^58^, was reduced in experimental nests, perhaps as a consequence of their reduced begging^46^. Our manipulation also affected prothonotary warblers, but in a different manner, as offspring condition in experimental nests was maintained at the same level as for control offspring, on average, but the number of young fledged per egg laid was reduced relative to control nests. Thus, our results suggest that increasing environmental temperature during incubation may affect the classic trade-off parents face between the size and number of offspring^60^.

We also detected species-specific effects of nest microclimate on posthatching parental care. Similarly, Auer and Martin^27^ detected species-specific effects of egg cooling during the first five days of incubation on posthatching parental care and nestling growth. Egg size per se may also affect heat loss during incubation off-bouts; thus, between-species differences in egg size might account, at least in part, for the effects we observed. However, we also found in the current study that posthatching parental behaviour changed with the increase in nest-cavity temperature, as evidenced by an interaction between treatment and species in their effect on postnatal maternal care. We expected that differences between species in nest construction might generate species-specific effects, as Carolina wren clutches are relatively well-insulated and prothonotary warbler clutches are not insulated at all during incubation off-bouts. We also detected a species-specific treatment effect on the probability that parents would breed again in the study population in subsequent years. Collectively, these species-specific effects highlight the importance of considering differences between species in their responses to experimental manipulations in wild populations, and the need to replicate both experimental and observation findings across species and contexts.

Although increased mass is predictive of survival, some studies suggest that reductions in body mass might actually be beneficial under gradually increasing temperatures^61–63^. Presumably, a smaller body dissipates heat more rapidly than a larger body because of the increased ratio of body surface area to volume. Thus, even if being in poorer condition tends to reduce survival, all else being equal, warmer environments may favour individuals who can more easily dissipate heat, although evidence for this is mixed^64^. For the Carolina wren, lighter offspring may be better suited to warmer environments, and a reduction in maternal brooding time may indicate that warmer temperatures allow females to invest relatively more energy toward self-maintenance. Whether these hypotheses are true awaits further study. It also must be acknowledged that the patterns we observed in this study are derived from a single year. As such, we advise caution in generalizing our results to other contexts. Nonetheless, the environmental conditions experienced during the breeding season were not particularly anomalous, so we consider it unlikely that the patterns we report are a response to unusual environmental conditions. We also note that the underlying mechanisms for the effects we observed remain unclear. However, the observations reported in the current study raise novel hypotheses surrounding the mechanisms generating carry-over effects, including whether those we observed here were manifest directly on offspring or indirectly through posthatching parental behaviour. Future work will disentangle these components and shed light on the contribution of maternal incubation behaviour to carry-over effects (i.e., increased temperatures during on-bouts vs. a reduced rate of egg cooling during off-bouts), and the underlying links between environmental temperature and nestling begging and posthatching growth and the costs of parental care.

In conclusion, both incubation duration and the nestling period were shortened under increased environmental temperature. In addition to changes in the timing of developmental stages, we detected carry-over effects on offspring phenotype, including their begging behaviour, and on posthatching survival. Collectively, our results suggest that the ability of different species to buffer the effects of rapid environmental change on fitness may be limited. Future work will continue to shed light on how changing temperatures affect the various life-history stages in wild populations.

## Methods

### Study species and field site

Each of our study species is insectivorous and generally nests in preformed cavities. Only the female incubates eggs and broods young nestlings, but both parents provision young after hatching^65,66^. Carolina wrens (18–23 g) are year-round residents of the Southeastern United States, with peak egg production from April through most of July^66^. Incubation lasts 13–18 d^67^, and fledging occurs 10–16 d after hatching^66^. Prothonotary warblers (14–16 g) are neotropical migrants, breeding in the eastern United States and wintering in the West Indies and Central America, and northern South America. Males arrive on the breeding grounds from late March through April, followed shortly thereafter by females^65^. The warblers breed in Tennessee from the end of April through July; ca. 50–75% of females attempt a second brood after successfully fledging a first brood, with peak egg production occurring in May for the first brood and late June and early July for the second^65^. Incubation lasts 12–14 d, and fledging occurs 9–11 d posthatching^65^.

This study was conducted at the Edward J. Meeman Biological Station (35.363°N, 90.017°W) in west Tennessee, USA. We established nestboxes (*N* = 220) for breeding wrens and warblers before the 2017 field season within secondary deciduous and mixed forest habitat on the Loess Bluffs above the Mississippi Alluvial Plain. Nestboxes (interior dimensions: 14.0 cm × 10.2 cm (length × width) basal area, with a pitched roof 14.0 cm high in the front and 15.4 cm high in the back of the nestbox) had a wide slot entrance (10.2 cm wide × 4.4 cm tall) beneath an eave of 3.8 cm, and were distributed on an 80–90 m grid over ca. 100 ha of forest.

### Field procedures

We checked nestboxes at least twice weekly for the formation of new nests. Within each species, a coin flip determined the first treatment, and treatments were alternated with each new nest thereafter. The number of nests within each treatment was evenly represented (*N* = 48 nests; Carolina wren: 9 control and 8 experimental nests; prothonotary warbler: 15 control and 16 experimental nests). All nestboxes had a resistive film heater (Adafruit Industries product 1481) attached to the inside rear wall once the clutch was complete and incubation was underway (i.e., the first full day of incubation with a complete clutch). In both experimental and control nests, heaters were wired to a small battery pack outside the nest. Heaters were powered by four rechargeable AA batteries, which were replaced each morning during the period of the manipulation (each day of incubation) and heated nests for ca. 4–6 hr each day. Control nests contained a heater and battery pack, and were visited by us with the same frequency as experimental nests, but the heaters were not turned on.

All nests were equipped with two thermochron iButton dataloggers to record internal and external ambient temperature. Data loggers that measured internal temperature were located midway up the sidewall of the nestbox while external data loggers were placed underneath the nestbox to obtain shaded ambient temperature. Our objective was to manipulate environmental temperature, which could affect embryos indirectly through effects on maternal behaviour, and not to manipulate egg temperature *per se*. Thus, we used iButtons to assess the efficacy of our manipulation of environmental temperature rather than attempt to estimate egg temperature. Indeed, if females modulate incubation behaviour according to environmental temperature, then changes in female incubation behaviour that ultimately result in stable egg temperatures would preclude our ability to assess environmental temperature. As expected, the manipulation caused a subtle, yet noticeable, increase in environmental temperature and carry-over effects after hatching (see Results).

Four days into incubation, we video recorded nests to document female incubation behaviour (incubation constancy, number of on-/off-bouts) during the time at which heating coils were actively heating nests. We used a digital video recorder on a 1.5-m pole ca. 1 m from the nestbox, which was placed the day before the recording so the birds would be habituated to its presence. Observations lasted an hour following resumption of normal parental activities, which provides a representative sample of consistent individual differences in parental behaviour^68^. We define incubation constancy as the proportion of time the female is within the nestbox during the observation, and each visit to the nest as an on/off bout^69^. We captured adults at the nest either during the second half of incubation or while provisioning nestlings by either capturing them inside nestboxes or using mist nets placed outside the box. Upon capture, adults were banded with a unique U.S. Geological Survey (USGS) aluminium leg band. Both female and male wrens, and male warblers, were also banded with 3 additional coloured leg bands arranged in unique combinations so they could be visually identified and observed without capture.

Once eggs hatched, we subsequently monitored nests until fledging. Four days after hatching, which corresponds to the period of most rapid growth in each species, we observed parental provisioning using digital videos as described for incubation behaviour above. During these observations, blind with respect to treatment, we recorded food deliveries by the male and female parents, faecal sacs removed by parents, and, for females, the amount of time she spent in the nest brooding her young, which can often have a positive effect on nestling growth and size^70^. At this time, we also recorded nestling begging vocalizations using a microphone inside the nest with a digital voice recorder outside the box^71,72^, and we used Raven Pro 1.5 sound analysis software (Cornell Lab of Ornithology) to count the begging vocalizations^72^. All nestlings were processed prior to fledging, during which time they were weighed (0.1 g) and had their tarsus length measured (0.1 mm) using dial callipers to obtain a measure of skeletal size. Nestlings were banded with a unique USGS leg band for individual identification, and weighed for the last time at different ages for the two species to reflect pre-fledging mass; Carolina wrens were banded at 9 days and prothonotary warblers at 8 days posthatching (where hatching date is the day on which hatching begins for a given nest); body mass prior to independence is often a strong predictor of the subsequent recruitment of offspring as breeding adults into local populations^58,73–75^. We subsequently visited nests daily after banding to check for fledging.

Of the prothonotary warbler adults in our study, 56% returned to breed the following year (23 of 45 marked adults in 2017 returned to breed in 2018); a return rate consistent with results reported for multi-year studies of other populations^76^. Carolina wren males were much more difficult to capture than females, especially after having been captured previously. However, we identified all females breeding in our nestboxes in 2017 and 2018, and found that 65% of the female wrens breeding in 2017 (11 of 17 females) also bred in 2018. Returning adults in both species typically settled in close proximity to the nestboxes they used in 2017.

### Statistical analyses

All analyses were conducted using SAS (ver. 9.4), with two-tailed hypotheses (α = 0.05), and sample sizes may differ for some analyses because of missing data (e.g., because of nest failure). When assessing treatment effects, we initially included a treatment × species interaction, but removed this interaction when non-significant. We first analysed the temperature of nest microclimates using both the raw internal temperature of a nestbox and the difference between internal temperature and shaded ambient temperature outside the nestbox. We assessed these using nested ANOVAs (PROC MIXED) with treatment as a fixed effect and nest within treatment as a random effect to account for the non-independence of observations within the same nest. We tested for an effect of our treatment on maternal incubation behaviour while heating coils were active, assessing this using a multivariate ANOVA (MANOVA; PROC GLM) with incubation constancy and the number of on/off bouts per hour as dependent variables, with treatment and species as crossed fixed effects, and we also included clutch-initiation date as a covariate to control for environmental variation. We also ran a similar model with environmental temperature as a continuous predictor in the place of treatment to assess whether natural variation in environmental temperature in general (i.e., average daily temperature during incubation) influenced maternal incubation behaviour. As a follow-up to our multivariate analyses, we calculated standardized canonical coefficients that summarize the strength of the relationship between predictor and dependent variables; namely, when a given predictor is statistically significant, the absolute value of standardized canonical coefficients indicates the extent to which its associated dependent variable is contributing to the effect^77,78^.

We assessed effects of our treatment on the duration of the incubation and nestling-rearing stages using a MANOVA with these two dependent variables, and included treatment and species as crossed main effects with clutch-initiation date as a covariate. We then tested for an effect on hatching success (i.e., the proportion of eggs laid that hatched) using a linear model (PROC MIXED) with treatment and species as crossed main effects and clutch size as a covariate. We analysed nestling begging vocalizations on day four posthatching using a linear model with treatment and species as crossed main effects, and hatching date and brood size as covariates. We then included these nestling begging vocalizations as an independent variable in a MANOVA assessing parental care. To assess parental care, we used maternal (i) provisioning rate (feeding trips per hour) and (ii) time spent brooding her young per hour, and (iii) male provisioning rate as dependent variables in a MANOVA with treatment and species as crossed main effects, and offspring begging frequency as a covariate.

We then tested for an effect of temperature treatment on the proportion of young fledged, including treatment and species as crossed main effects, using a generalized linear model (PROC GLIMMIX) with the number of young fledged as the dependent variable and clutch size as the binomial denominator (i.e., successes/trials syntax) to account for the number of young fledged per egg laid^79^. We then analysed nestling pre-fledging body mass using a linear mixed model with nest as a random effect, and we included treatment and species as crossed main effects with the day of the year on which nestlings were banded and weighed as a covariate; we also included tarsus length as a covariate to control for skeletal size, such that our analysis is reflective of size-adjusted body mass, or condition^80^. Finally, we assessed potential long-term costs to parents’ probability of breeding within their study populations in the subsequent year in relation to our treatment and variation in parental care, in addition to a treatment × species interaction. We did this using a generalized linear model (PROC GENMOD) with a binary response (bred the next year or did not). We included several covariates related to parental care, including the mass of offspring prior to fledging and parental responsiveness to nestling begging signals. We assessed parental responsiveness to nestling begging as the residual of a provisioning rate × begging rate regression, so parents with increased values delivered more food to their young than would be expected from the intensity with which their young solicited food. We also included brood size and an interaction between provisioning effort and brood size, as parental return rates often decline with reductions in fledging success, likely because of dispersal following a disappointing cycle^76,81,82^.

### Ethical note

All research activities were performed in accordance with current laws of the United States and ABS/ASAB guidelines for the ethical treatment of animals. All activities were approved by the University of Memphis Institutional Animal Care and Use Committee, USGS banding permit 24052, and Tennessee Wildlife Resources Agency Scientific Collection Permit 3950.

## Supplementary information

Supplementary information

Dataset 1

## Data Availability

All data generated and analysed for this study are readily available upon request from the corresponding author.
