# Supplementary material for: Nest microclimate during incubation affects posthatching development and parental care in wild birds
Source: Sci Rep. 2019 Mar 26;9:5161. doi: 10.1038/s41598-019-41690-4 (PMC6435697; doi:10.1038/s41598-019-41690-4)
Supplement: Supplementary file 1 — Supplementary information [file 41598_2019_41690_MOESM1_ESM.docx]

**Supplementary information**

**Manuscript title:** Nest microclimate during incubation affects posthatching development and parental care in wild birds

**Authors:** Alexander J. Mueller, Kelly D. Miller, E. Keith Bowers


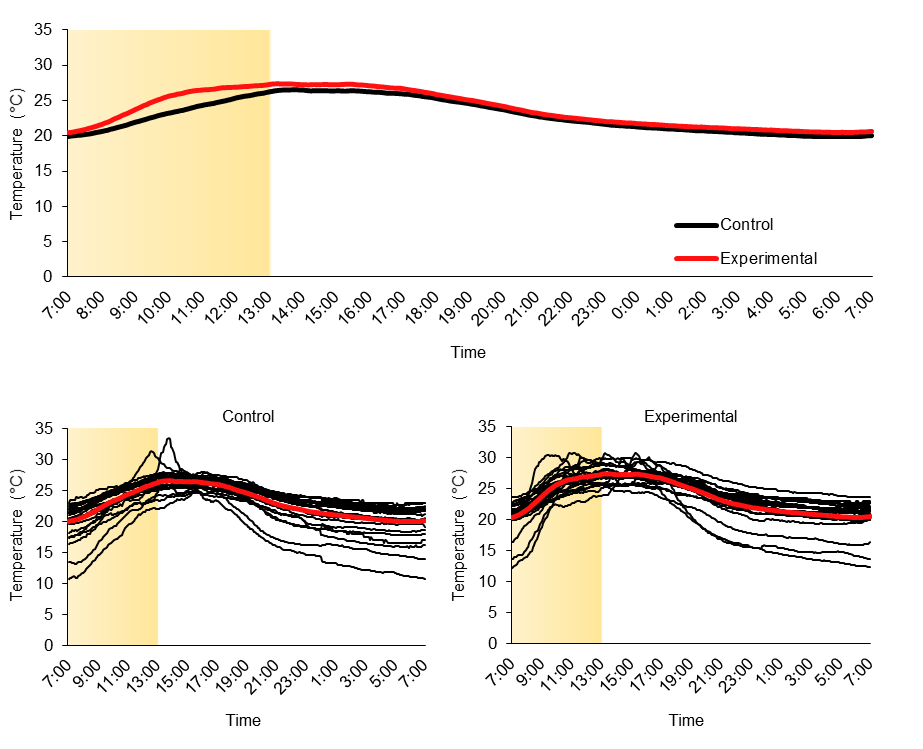


**Figure S1.** Internal iButton temperature measurements. The top panel compares overall treatment means for all nests on each day of incubation averaged over 24 h. In the lower two panels, black lines represent reaction norms for individual nests, calculated as the mean from each day of incubation within a given nest; red lines represent mean temperature values for that treatment overall. The shaded area depicts the time when most heaters would have been active for experimental nests.


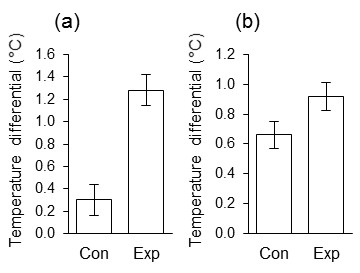


**Figure S2.** Temperature differentials of internal and external temperature measurements for each treatment. (a) Cropped data set only comparing temperatures between 7:00-13:00, (b) Full data set comparing all temperature records during incubation. Plotted are least-squares means ± SE.
